# Metabolic Footprints and Molecular Subtypes in Breast Cancer

**DOI:** 10.1155/2017/7687851

**Published:** 2017-12-24

**Authors:** Vera Cappelletti, Egidio Iorio, Patrizia Miodini, Marco Silvestri, Matteo Dugo, Maria Grazia Daidone

**Affiliations:** ^1^Department of Applied Research and Technical Development, Fondazione IRCCS Istituto Nazionale dei Tumori, Milano, Italy; ^2^Core Facilities, High Resolution NMR Unit, Istituto Superiore di Sanità, Rome, Italy

## Abstract

Cancer treatment options are increasing. However, even among the same tumor histotype, interpatient tumor heterogeneity should be considered for best therapeutic result. Metabolomics represents the last addition to promising “omic” sciences such as genomics, transcriptomics, and proteomics. Biochemical transformation processes underlying energy production and biosynthetic processes have been recognized as a hallmark of the cancer cell and hold a promise to build a bridge between genotype and phenotype. Since breast tumors represent a collection of different diseases, understanding metabolic differences between molecular subtypes offers a way to identify new subtype-specific treatment strategies, especially if metabolite changes are evaluated in the broader context of the network of enzymatic reactions and pathways. Here, after a brief overview of the literature, original metabolomics data in a series of 92 primary breast cancer patients undergoing surgery at the Istituto Nazionale dei Tumori of Milano are reported highlighting a series of metabolic differences across various molecular subtypes. In particular, the difficult-to-treat luminal B subgroup represents a tumor type which preferentially relies on fatty acids for energy, whereas HER2 and basal-like ones show prevalently alterations in glucose/glutamine metabolism.

## 1. Molecular Subtypes in Breast Cancer: A Major Step towards Treatment Prediction

In light of the progress achieved in early diagnosis, surgery, chemotherapy, and endocrine therapy in breast cancer, there is no doubt that the biological heterogeneity of this tumor remains the major obstacle on the way towards an optimal disease control. Such heterogeneity, which has been recognized some time ago, has been initially ascribed to hormonal milieu (menopausal status), in times when studies on hormone sensitivity were still in their infant state. Later, attempts by Jensen and Jordan [1] and Sledge and McGuire [2] to distinguish breast tumors by their ability or their lack of ability to bind 17*β*-estradiol with high affinity, limited capacity, and high specificity gave a biological and molecular basis to the clinically well-recognized fact that certain tumors were hormone-sensitive whereas others were not [3]. Several evidences were being collected in the meantime, showing that proliferative activity, which varies greatly among individual tumors, may provide [4] an explanation for both the variable natural history of the disease and for distinct sensitivity to anticancer agents.

During the following years, with the gradual recognition that breast cancer is definitely not a single disease, but rather a group of diseases characterized by clinical, morphological, and molecular heterogeneity, the description of the subtypes of breast tumors has become more and more sophisticated. Furthermore, after the advent of microarray techniques, a prominent role in defining the landscape of breast tumor was played by gene expression studies [5]. Nowadays, at least four molecular subtypes are commonly recognized: luminal A, luminal B, HER2-enriched, and basal-like (roughly corresponding to the so-called triple-negative breast cancer, TNBC) to which the categories of claudin-low and normal-like can also be added. Also, integrated analysis of copy number alterations with gene expression analysis further extended the number of subtypes to ten [6]. Nonetheless, even when analyzing large case series for different molecular features (microRNA/methylation/copy-number alterations/gene expression = PAM50 and reverse-phase proteomic analysis), the four main subgroups defined by gene expression classifiers [7] still recapitulate most of the heterogeneity [8]. Despite some heterogeneity within the luminal subgroup, this categorization (by molecular signatures or by pathological surrogates) presently is the only tool available for treatment guidance in women with early-stage invasive breast cancer approved by the ASCO [9] and by the St. Gallen [10] guidelines.

On one side, tumor molecular subtypes do in fact recapitulate the presence or absence of specific drug targets such as the estrogen receptor and the cell membrane growth factor HER2, but on the other side, they also underscore a complete different prognostic landscape. If we assume with a certain approximation (due to the exclusion of contribution by immunity) that, as far as concerns the natural disease history, the major prognostic driver is proliferative activity, luminal tumors are put into the most favorable position, with HER2-enriched and basal-like on the opposite end. However, the availability of target treatments, that is, endocrine therapy and HER2-targeting drugs, has completely modified the scenario offering to women bearing HER2-enriched tumors an advantage with respect to those with basal-like tumors who are instead affected by a target-orphan disease.

The relevance of proliferation does not rely only on its prognostic value but also derives from the fact that chemotherapy targets highly proliferating cells and is ineffective on quiescent cells. This clearly makes basal tumors more chemosensitive compared to luminal A tumors, though it does not revert their poor survival probability and this category urgently needs additional treatment targets. In the luminal disease instead, as underlined by the St. Gallen International Consensus Panel in 2015, major concerns regard the identification of the most difficult to treat category, namely, luminal B tumors, by simply applying a cutoff to a marker such as Ki-67 which shows a continuous distribution.

Apart from low expression of proliferation and cell cycle-related genes, luminal A tumors are distinguished by higher expression of *PR* and *FOXA1*, *GATA3*, and *XBP1*, whereas the *ESR1* gene is expressed at comparable levels as in luminal B tumors [5], [11]. Their mutational rate is lower compared to other subtypes, and the most frequently reported mutations relate to *PIK3CA*, *GATA3*, and *MAP3K1*. Luminal A tumors are characterized by low histological grade and are diploid in contrast to luminal B tumors which are high-grade and frequently aneuploid. Recommended treatments reflect the molecular asset of such tumors as hormone therapy is suggested for luminal A tumors whereas for luminal B chemotherapy and anti-HER2 therapy (when *HER2* is highly expressed or amplified) are additional options.

HER2-enriched breast tumors are characterized by high expression of *ERBB2* at the RNA and protein level and by increased levels of genes coamplified with *ERBB2* such as typically *GRB7*. Such tumors express luminal genes at an intermediate level and do not express or express at low levels basal-related genes such as *KRT5*. Mutation frequencies are high among HER2-enriched tumors and include mainly *TP53* and *PIK3CA*.

In the basal-like subgroup of patients, no specific targets are available yet and chemotherapy remains the only option. Such tumors are highly proliferating, mostly aneuploid and high-grade and besides expressing the basal keratins (*KRT5* and *KRT6*) often express high levels of *EGFR*, present complex genomic rearrangements, and often harbor *TP53* mutations. Interestingly, these latter tumors represent such a peculiar type of breast tumors that appear to be more similar to squamous cell lung cancer rather than to luminal breast cancer. Bladder tumors also include a distinct molecular subtype with features very similar to basal-like breast cancer [12, 13].

The combined clinical and molecular heterogeneities in breast cancer urgently call for the identification of additional subtype-specific treatment targets beyond the classic steroid hormone receptors and HER2 since somatic mutations in actionable genes such as *PIK3CA* represent a possibility only for a limited percentage of patients. In such a context, the metabolic peculiarities of tumor cells, especially in those molecular subtypes such as luminal B and basal-like tumors where treatments still pose some difficulties, could represent an innovative way for improving and personalizing treatment outcome.

## 2. Metabolism: A Long-Standing Hallmark of the Cancer Cell

Despite molecular heterogeneity, certain metabolic features tend to be distinguishable in tumor tissues in comparison to normal tissues since a reprogramming of metabolism is necessary for cancer cell proliferation and survival within their environment. Such metabolic rewiring provides cancer cells with (i) the rapid generation of energy in terms of (ATP); (ii) increased synthesis of biochemical building blocks for lipids, carbohydrates, proteins, and nucleic acids; and (iii) proper redox potential and stability [14, 15].

In fact, following the introduction of altered energy metabolism to the list of cancer hallmarks [16], there are several evidences on a wider metabolic rewiring in cancer cells, which not only includes cellular bioenergetics but also a more complex network of deregulated biochemical pathways associated with altered signaling pathways essential to tumor proliferation, growth, and invasion. Such a metabolic plasticity adopted by cancer cells allows counteracting the host defense and eventually resists the attack of anticancer treatments. Consistently, advanced bioinformatics analyses have highlighted that mutations, deletion, and amplifications affect not only crucial signaling pathways but also metabolic pathways that are determinant for tumor growth and response to cancer therapy.

**Figure 1:**
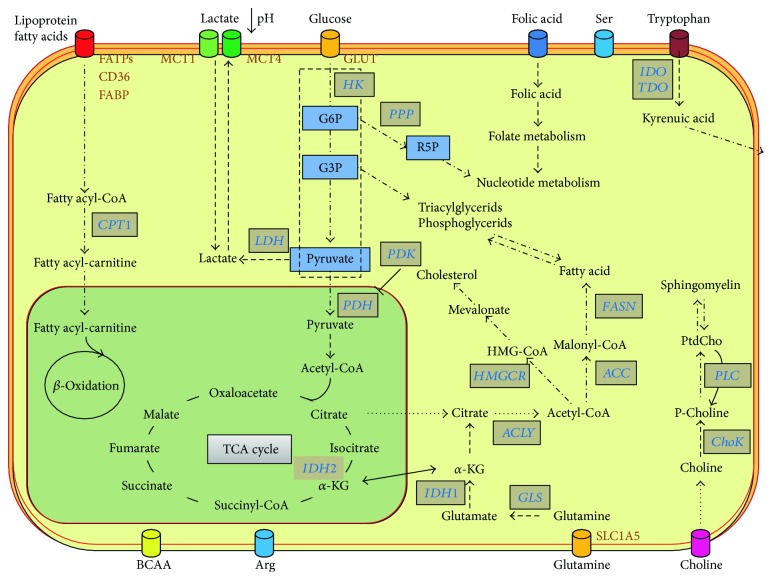
Schematic overview of altered metabolism in cancer.

The first evidence for an altered metabolism in tumors regards the glucose metabolism and was reported by Warburg et al. [17], who described a shift away from an oxidative towards a glycolytic energy metabolism (even under aerobic conditions) to produce the ATP necessary for proliferation. This metabolic shift, known as *Warburg effect*, can be observed regardless of oxygen availability. In such a scenario, several evidences have clearly shown that transcriptional factors such as HIF, c-Myc, and p53 are able to modulate the expression and activities of glucose transporters and of enzymes involved in the glycolysis and pentose phosphate pathways (PPP) or in the tricarboxylic acid (TCA) cycle [18]. The PPP (parallel pathway of glycolysis, starting from glucose-6-phosphate) is crucial for generating important biomolecules such as NADPH and ribose sugars. The NADPH is essential for various metabolic requirements such as ATP production, biosynthesis of lipids, and for counteracting oxidative stress. Instead, the ribose sugar is essential information of an intracellular pool of nucleosides for proliferating cells. In fact, a high ratio between the oxidative and nonoxidative branches of PPP promotes the proliferation in several types of cancer cells [19]. Thus, in both glycolysis and PPP, precursors and substrates for macromolecules like nucleic acids, lipids, and proteins are generated to support overall cancer growth. A schematic picture of metabolic alterations in cancer is shown in Figure 1.

Besides glucose, glutamine is essential for the increasing demands of ATP and lipids. Tumor cells employ glutamine not only as carbon donor but also as a nitrogen donor for amino acids and nucleotide biosynthesis and for the formation of *α*-ketoglutarate (*α*-KG), involved in ATP production in mitochondria. Glutamine can enter the cell through glutamine transporters like ASCT2 and SLC38A5. Transcription factors such as c-myc upmodulate the expression of the ASCT2 transporter and regulate the expression of other glutamine transporters and enzymes involved in the conversion of glutamine to glutamate (GLU) such as glutaminase (GLS1) [20]. Lactate-induced c-Myc activation triggers the expression of glutamine transporter ASCT2 and of GLS1, resulting in enhanced glutamine uptake and catabolism in tumor cells [21].

In addition to glutamine, metabolism of other amino acids such as glycine and serine and of the branched chain amino acids leucine, isoleucine, and valine could play an important role in cancer metabolic phenotype and tumor microenvironment [22]. Serine and glycine are biosynthetically connected and are essential to the synthesis of all macromolecules, such as proteins, lipids, and nucleic acids, used in cellular growth and proliferation. These two amino acids participate to a complex cyclic metabolic network of folate metabolism, known as one-carbon metabolism crucial for nucleotide synthesis, methylation, and reductive metabolism [23, 24]. Indeed, upregulation of serine/glycine metabolism is associated to cancer cell proliferation and to poor prognosis in patients [25]. Glycine is also an integral element of the main antioxidant tripeptide glutathione, and it thus regulates the redox balance of the cells.

In recent years, there has been a strong interest in understanding tryptophan and L-arginine biochemistry, and particularly their catabolic pathways, which often are deregulated in cancers. Tryptophan is involved in the modulation of immune tolerance and in the suppression of antitumor immune responses [26, 27]. The catabolism of tryptophan occurs both via indoleamine-2,3-dioxygenase (IDO) and tryptophan-2,3-dioxygenase (TDO) and conversion into kynurenine or by tryptophan hydroxylase-1 (TPH-1) into tryptophan to 5-hydroxytryptophan (precursors for serotonin biosynthesis) [26, 27]. IDO is expressed by both immune cells and tumor cells [28, 29]; IDO-expressing dendritric cells subtract this amino acid from the extracellular medium limiting tryptophan supply to surrounding T cells. In this way, the depletion of tryptophan and the accumulation of immunosuppressive tryptophan catabolites do impair T cell activation and proliferation inducing anergy and apoptosis [30].

Several types of tumors have abnormalities in their arginine metabolism enzymes. This nonessential amino acid participates in different pathways that include urea cycle, polyamine, and nitric oxide synthesis. L-arginine could have pleiotropic effects by modulating T cell metabolism potentiating their survival and antitumor activity [31]. In addition, enzymes of arginine metabolism such as nitric oxide synthase (iNOS) and arginase (ARG) could create toxic reactive nitrogen species which induce apoptosis in lymphocytes and modulate tyrosine phosphorylation of several proteins leading to downregulation of membrane receptors such as CD4, CD8, and chemokine receptors in T cells [26].

Other metabolic hallmarks of cancer cells include aberrant choline phospholipid and lipid metabolism [32–34]. Different studies have reported a strong lipid and cholesterol avidity in highly proliferative cancer cells by activating the uptake of exogenous (or dietary) lipids and lipoproteins or by enhancing de novo lipid and cholesterol biosynthesis starting from cytosolic acetyl-CoA [34]. Lipid de novo biosynthesis involves a multiple step process with a conversion from acetyl-CoA to malonyl-CoA by the acetyl-CoA carboxylase (ACC). The subsequent condensation reactions catalyzed by fatty acid synthase (FASN) lead to saturated fatty acids, where the degree of unsaturation could be induced by specific stearoyl-CoA desaturase (SCD). Elevated FASN expression was indeed reported for breast, prostate, and other types of cancer [35], and, as in the glucose metabolism, the lipid biosynthetic enzymes are under strict control of cellular signaling such as PI3k/Akt [36, 37].

Fatty acid oxidation (FAO) occurs mainly in mitochondria and is responsible for the breakdown of long-chain acyl-CoA to acetyl-CoA. This multistep process is regulated at the transcriptional level by PPARs, SREBP1, and PGC-1*α* and at the posttranscriptional level by ACC, malonyl-CoA decarboxylase (MCD), and carnitine O-palmitoyltransferase 1 (CPT1) regulation. The long-chain acyl-CoA enters the fatty acid *β*-oxidation pathway, which results in the production of acetyl-CoA, NADH, and FADH2 from each cycle of FAO and subsequent mitochondrial ATP production. FAO offers more energy (ATP) as compared to carbohydrates and generates intermediates that could stimulate cancer cell proliferation and survival. In the last years, new evidences highlighted that acetyl-CoA generated by FAO could be converted into citrate acetyl-CoA which enters the Krebs cycle to produce citrate, which can be exported to the cytoplasm to engage NADPH-producing reactions [38] and can act against oxidative stress and xenobiotics and allow cancer proliferation and survival [39]. Accordingly, intracellular accumulation of neutral lipids (triacylglycerol and cholesteryl esters) is now considered as a hallmark of cancer aggressiveness [40–43].

Phospholipids not only are the basic structural components of membranes but also represent reservoirs of second messengers for reactions involved in key regulatory functions of mammalian cells. Phosphatidylcholine (PtdCho) and phosphatidylethanolamine (similarly to phosphatidylinositol, Ptdlns) can generate second messengers such as diacylglycerol (DAG) and phosphatidic acid, which in turn is a precursor of DAG, lysophosphatidic acid, and arachidonic acid, through three major catabolic pathways, respectively, mediated by specific phospholipases of type C (PLC) and D (PLD), acting at the two distinct phosphodiester bonds of the phospholipid headgroup and by phospholipase A2 (PLA2) in the deacylation reaction cascade [33]. Phosphocholine, either produced by choline kinase (ChoK) in the first reaction of the three-step Kennedy biosynthetic pathway or via PLC-mediated PtdCho catabolism, has also been shown to be mitogenic, by acting as a mediator in growth factor-promoted cell proliferation [32, 33]. Several relationships exist in fact between the PtdCho cycle and cell receptor-activated signal transduction pathways with implications regarding the biogenesis and utilization of other lipids and phospholipids [33, 44].

There are evidences that metabolic reprogramming includes not only activation or inhibition of specific metabolic pathways but also posttranslational modifications where specific metabolites (lysine methylation and acetylation, glycosylation, palmitoylation, and S-glutathionylation) are covalently bound to proteins. These modifications are responsible for cell proliferation, differentiation, migration, and altered signal transduction [45].

Aberrant glycosylation represents a potential hallmark of oncogenesis [46, 47]. These alterations consist in (a) changes in the amount, linkage, and acetylation of sialic acids; (b) modification of proteins by the monosaccharide *N*-acetylglucosamine (*O*-GlcNAcylation); (c) alterations in sulfation of glycosaminoglycans; and (d) modulation of the enzymes that attach glycosylphosphatidylinositol (GPI) anchors to proteins [48–50]. The enzymes responsible for these alterations are regulated by oncogenic growth factor signaling and represent novel therapeutic targets for cancer diagnostic and therapeutic strategies, such as the development of glycosyltransferase inhibitors, glycomimetics, and glycan/glycopeptide-based vaccines [51].

## 3. Metabolomics and Breast Cancer

Metabolomics represents the last addition to a bunch of promising *omic* sciences such as genomics, transcriptomics, and proteomics, whose contribution to understanding cancer biology and to guiding treatment is unquestionable. As described above, biochemical transformation processes underlying energy production and biosynthetic processes have been recognized as a hallmark of the cancer cell and hold a promise to build a bridge between genotype and phenotype. Since the term breast tumors represents a collection of different diseases, understanding metabolic differences between molecular subtypes could offer a way to identify new subtype-specific treatment strategies, especially if metabolite changes are evaluated in the broader context of the network of enzymatic reactions and pathways.

Metabolic alterations in breast cancer have been studied for many years applying different techniques spanning from the less sensitive, but nonsample destructive nuclear magnetic resonance- (NMR-) based approaches, including high-resolution magic angle spinning (HR-MAS) in intact tissues, to the more sensitive and specific liquid chromatography (LC), gas chromatography (GC), and mass spectrometry- (MS-) based approaches. All studies (for a comprehensive review see [52]) report differences between tumor versus nontumor tissues [53–57].

Although, depending on the specific approach, the metabolites showing different levels between tumor and nontumor tissue may vary, a core of metabolites, namely, glycine, taurine, phosphocholine, and lactate, is consistently upregulated in tumor samples with respect to normal samples. By using gas chromatography time-of-flight mass spectrometry (GC-TOF-MS) for developing a signature of 13 metabolites upregulated in cancer samples and 7 metabolites upregulated in normal samples, Budczies et al. [58] could separate cancer from normal samples with 95% sensitivity and 94% specificity. A clear distinction of tumor from normal samples was also achieved using HR-MAS which enables the analysis of intact tissues and is sufficiently rapid for allowing distinction of tissues in the operation theatre [59]. Metabolomics, however, not only allows a distinction between tumoral and normal samples, but it is also suitable for differential diagnosis of benign versus malignant lesions [54, 56] and, by focusing on selected tumor metabolic markers, namely, phosphocholine, lactate, and lipids, even correlations with histological grade were reported [55].

Metabolomic studies not only confirm differences among breast cancer subgroups defined by molecular, histological, or clinical data but also have the potential to further extend classifications, offering this way additional clinical value. This concept is clearly supported by data derived from integration between HR-MAS MRS and gene expression microarrays performed on 46 early breast cancer patients [60]. Metabolomic analysis allowed subtyping of luminal A breast tumors into three distinct groups differing for the contents of *α*-glucose, *β*-glucose, amino acids, myo-inositol, and lipid residues. In particular, one subgroup-denominated A_2_ characterized by lower glucose levels and higher alanine levels included tumors with increased glycolytic activity. Such type of studies supports the concept that metabolomic profiling of breast tumors could represent an additional layer, worth to be explored, in our search of increasingly personalized treatment approaches.

However, despite potentialities, metabolomics retains several intrinsic limitations, which have so far substantially prevented its widespread implementation in the clinical setting. Major limitations, deriving from both biological and experimental factors, include interindividual differences among patients, sampling variability, and a substantial lack of validated protocols for tissue handling. Moreover, biological factors such as warm and cold ischemia may have a crucial impact on the results of omics-based investigations. The effects of the time spent by a tissue specimen under conditions of warm ischemia induced by vessel ligation and resection from the body can be hardly predicted, as these effects depend upon the nature of the disease and the adopted surgical procedure. On the other hand, the effects of different times of cold ischemia (i.e., the time intervals from resection to fixation and/or to freezing for cryo-preservation) were investigated to ensure high-quality *omics* data. Recently, studies observed no significant changes in the content of individual metabolites in breast tumor samples frozen within 30 min of resection. After this time point, there were some metabolic changes in the content of phospholipid metabolites and in the levels of ascorbate, creatine, and glutathione [61]. A previous study by our group showed that under vacuum storage (UVS) tissue specimens for histological, transcriptomic, and proteomic examinations could be preserved up to 48 hours but that this method had limitations for metabolomic applications [62] since we found an increase in the free choline concentration in normal and tumor breast tissue under vacuum storage, indicating that the metabolome is more affected by the time of storage compared to other omics approaches.

## 4. The Milan Case Series

The breast cancer series from the Istituto Nazionale dei Tumori (INT) of Milan included 95 fresh-frozen samples from primary tumors obtained from women diagnosed with early breast cancer between the years 1990 and 1998. All patients were defined as axillary node-negative. For each sample, a written informed consent signed by the patient authorized the use of the tumor material leftover from diagnosis for research purposes. The study was approved by the INT Independent Ethics Committee and the local Institutional Review Board. Samples used for molecular studies were evaluated by a pathologist after evaluating percent of tumor cells on an adjacent section. Necrotic areas, fat, and normal tissue were carefully avoided, and samples were immediately snap-frozen in liquid nitrogen and then stored at −80°C until further use.

Gene expression profiling studies were performed by the INT Functional Genomics Core Facility on frozen samples using the Illumina platform (Sentrix Bead Chip HumanRef-6 v3, Illumina Inc., San Diego, CA). Samples were then stratified based on molecular subtype according to the expression of PAM50 genes [7] using two different approaches: unsupervised hierarchical clustering with Spearman's correlation as distance metric and average linkage and the nearest shrunken centroid method implemented in the pamr package [63]. In three cases, subtype attribution was discordant between the two methods and therefore only 92 samples with concordant molecular subtype labels (9 basal-like, 7 HER2, 36 luminal A, and 40 luminal B) were available for metabolite analyses. Raw and processed data were deposited to the Gene Expression Omnibus data repository with ID GSE104549. Samples' metabolomic analyses were performed by Metabolome Inc. (Durham, NC, USA). At the time of analysis, samples were extracted and prepared using Metabolome's standard solvent extraction method. The extracted samples were split into equal aliquots for analysis on the GC/MS and LC/MS/MS platforms.

In keeping with what stated above, and at difference to previous studies, the molecular classification of the tissue samples employed in this study was very robust and we therefore speculate that metabolic studies in our biological samples are likely to reveal new differences in metabolic pathways.

Globally, 408 compounds of known identity were identified in the samples. Paired comparison between the four molecular subtype categories revealed differences in biochemical levels among the categories as summarized in Table 1.

Raw data on metabolites reported in the figures are available in Supplementary file 1.

By simply comparing the numbers of biochemicals characterized by statistically significant different levels among categories, it appeared that the basal-like tumors represented the most distinct category from the metabolic point of view when compared to either luminal A or B tumors, as already observed from the transcriptomic point of view (see above), but showed less differences when compared to HER2 tumors. Luminal B tumors presented a different biochemical profile compared to luminal A, a finding which may have an important clinical relevance if such metabolites trace a targetable pathway. Metabolic changes are described hereafter in detail, referring to the metabolic main pathways disrupted in tumors (Figure 1).

In keeping with the differences observed for the levels of biochemicals, numerous metabolic pathways were altered among breast cancer molecular subtypes. In particular, there were significant differences in glucose utilization, TCA cycle metabolism, amino acid metabolism, membrane biogenesis, lipid oxidation, nucleotide catabolism, inflammation, and oxidative stress between the different classifications. Note that the extent of these changes was greatest in basal-like and HER-2-enriched tumors when compared to luminal A and B breast cancers.

### 4.1. Glucose Metabolism

**Figure 2:**
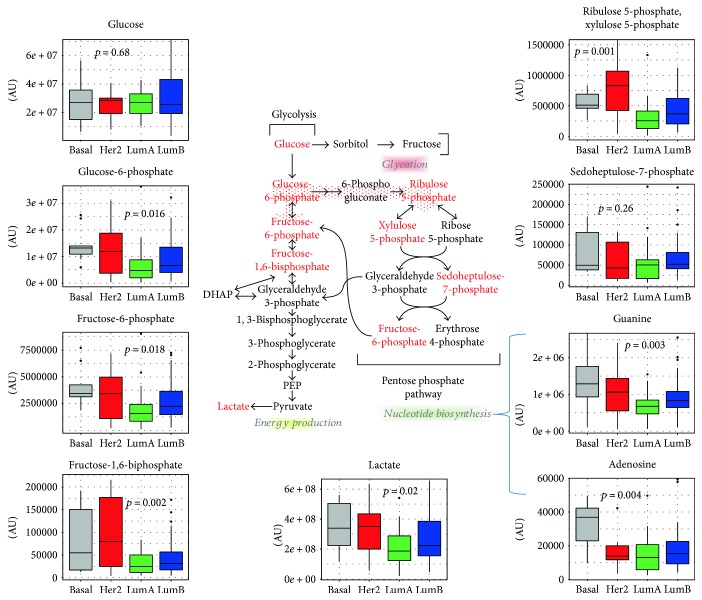
Glucose metabolism. Metabolites participating in the glycolytic and pentose phosphate pathways are schematically shown. For the metabolites written in red fonts in the scheme of the metabolic pathway, the levels across breast cancer molecular subtypes are reported as box plots. *p* values refer to one-way ANOVA on raw data. AU = arbitrary units.

Glucose, whose utilization is critical for the generation of cellular energy, nucleic acids, and biomass, presented similar levels between the various breast cancer subtypes (*p* = 0.68, One-way ANOVA), whereas for the downstream glycolytic intermediates, a net accumulation could be observed in luminal B, basal, and HER2-enriched tissues in comparison to luminal A (see Figure 2). Indeed, glucose-6-phosphate and fructose-6-phosphate levels were significantly different among subtypes (*p* = 0.02 and *p* = 0.02, resp.) increasing from luminal A to luminal B and to HER2-enriched samples with the highest difference between luminal A versus basal-like tumors (*p* = 0.03, *p* = 0.02). Fructose-1,6-bisphosphate (F1,6BP) levels also varied across the four molecular subtypes (*p* = 0.002, one-way ANOVA) with similar levels between luminal A and B tumors, but with significant increases between luminal A and basal-like, luminal A and HER2-enriched, and luminal B and HER2-enriched (*p* = 0.03, *p* = 0.007, *p* = 0.037, respectively, Tukey post hoc test). Interestingly, literature data also report the accumulation of F1,6BP showing that this glycolytic intermediate can directly bind to EGFR, which is highly expressed in basal-like/TNBC and this way enhance its activity. Indeed, in TNBC, the intermediate F1,6BP enhances lactose excretion, tumor growth, and immune escape [64].

Note that no differences in sorbitol (*p* = 0.69, ANOVA), fructose (*p* = 0.87, ANOVA), or the advanced glycation end product erythrulose (*p* = 0.45, ANOVA) were observed, suggesting that these tumors may immediately catabolize glucose for energy generation. Significantly different lactose levels were also observed (*p* = 0.024, ANOVA) across subtypes with a significant increase in basal-like versus luminal A tumors. Metabolite levels also confirmed the enhanced extent of glycolysis in basal-like and HER2-enriched tumors in comparison to luminal B tissues. However, since 3-phosphoglycerate (*p* = 0.51, ANOVA), 2-phosphoglycerate (*p* = 0.63, ANOVA), and phosphoenolpyruvate (*p* = 0.45, ANOVA) were similar between tumor subtypes; these observations may reflect shuttling of glucose-6-phosphate to PPP to generate NADPH, and pentose sugars and contribute to nucleotide biosynthesis.

The pentose phosphate metabolites, ribulose 5-phosphate and xylulose 5-phosphate (*p* = 0.001, ANOVA), were elevated in the more aggressive HER2-enriched molecular subtype (*p* = 0.002 luminal A versus HER2-enriched). These observations may be indicative of PPP flux facilitating the generation of nucleic acids as supported by elevated adenosine (*p* = 0.004, ANOVA) and guanine (*p* = 0.003, ANOVA) levels in basal-like tumors (Figure 2). Together, these findings suggest that glucose utilization was enhanced in all three subtypes (particularly in basal-like and HER2-enriched tissues) compared to luminal A breast cancer and are in agreement with evidence in the literature demonstrating that uptake of the glucose analogue fluordeoxyglucose F 18 (^18^F-FDG) in breast cancer patients correlates with their tumor proliferative potential.

Increased glucose utilization according to molecular subtype has already been reported in the literature. Accordingly, HER2 positive and TNBC mostly exhibit higher levels of glycolysis and consequently higher levels of expression of GLUT-1, the transporter responsible for membrane crossing by glucose [65, 66]. As the most invasive type of breast cancer, TNBC has the highest expression of GLUT-1 when compared to other subtypes [67, 68].

In keeping with our results, an increased activity of enzymes involved in glycolysis, like hexokinase and lactate dehydrogenase A (LDHA), has also been reported and linked to cancer cell [69, 70].

### 4.2. TCA Cycle

**Figure 3:**
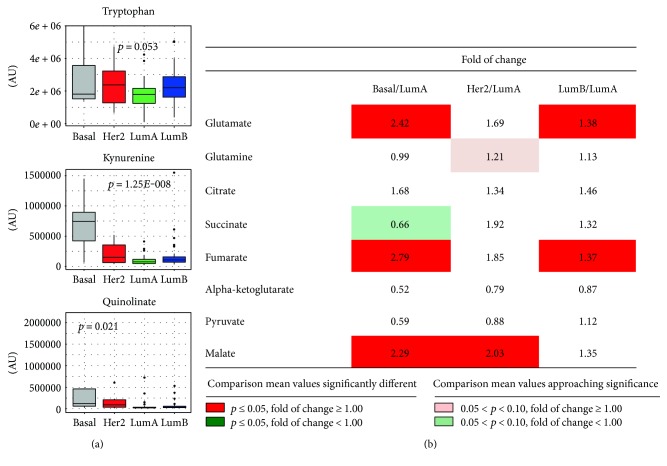
Amino acid metabolism and TCA cycle. (a) Box plots representing relative amounts of metabolites participating in the tryptophan-kynurenin pathway across molecular subtypes. *p* values refer to one-way ANOVA on raw data. (b) Relative differences in TCA metabolic intermediates across molecular subtypes. Data for basal-like, HER2-enriched, and luminal B tumors expressed as fold changes of raw data peak intensities with respect to the luminal A subtype are reported for each metabolic intermediate. Statistical significance of differences between mean values for each reported comparison was tested by the Welch *t*-test. Color codes refer to the range of calculated *p* values and to the direction of the differences between means, as detailed in the figure inset. AU = arbitrary units.

We next analyzed our data focusing on TCA cycle (Figure 3(b)). Starting from pyruvate levels (*p* = 0.78, ANOVA) and including other TCA cycle metabolites such as citrate (*p* = 0.64, ANOVA), alpha-ketoglutarate (*α*-KG) (*p* = 0.85, ANOVA), and succinate (*p* = 0.07 ANOVA), no statistically significant differences were observed across subtypes. However, at difference with the other TCA cycle intermediates, fumarate (*p* < 0.001, ANOVA) and malate (*p* = 0.001, ANOVA) levels showed statistically significant differences among subtypes, once again with increasing levels paralleling subtype-related aggressiveness. An excess of fumarate is well-described in tumors and may be linked to germline mutations in fumarate hydratase (*FH*), a condition which predisposes to hereditary leiomyomatosis and renal cell cancer [71], but interestingly also evidences for a link with *FH* are reported in breast and bladder carcinomas [72]. More generally, mitochondrion dysfunctions, either promoted by mutation in *FH*, isocitrate dehydrogenase (*IDH*), and succinate dehydrogenase (*SDH*) (seldom reported in breast cancer) or by other mechanism (for a comprehensive review see [73]), are described as possibly contributing to initiation and progression of cancer. Accordingly, both fumarate and 2-hydroxyglutarate (2-HG) levels appeared to be significantly different across subtypes (*p* < 0.001 and *p* = 0.01, respectively, ANOVA) and their levels were increased in more aggressive basal-like tumors compared to luminal ones (*p* = 0.004 in luminal A versus basal; *p* = 0.036 in luminal B versus basal for 2-HG levels; *p* < 0.001 in luminal A versus basal; and *p* = 0.001 in luminal B versus basal for fumarate). Indeed, 2-HG, succinate, and fumarate act as oncometabolites by inhibiting prolyl hydroxylases (PHD1-3) and stabilizing HIF-1*α*.

In particular, the accumulation of 2-HG is a well-known hallmark in cancer cells and is generally attributable to the occurrence of gain-of-function mutation in *IDH1* and *IDH2* [74]. Whereas, *IDH* mutation is reported as a very rare event in breast cancer [8, 75], literature data consistently report elevated 2-HG levels in about 50% of breast tumors [52]. This suggests that 2-HG accumulation in breast tumors is not mediated by IDH and may instead be mediated by MYC activation as suggested by Terunuma et al. [76].

Accumulation of 2-HG is linked with a DNA hypermethylation phenotype [74, 77], and it is reported that 2-HG is an inhibitor of *α*-KG–dependent enzymes including PHD1-3, histone demethylase KDM4C, and 5-methyl-cytosine hydroxylases TET2 [78]. DNA-methylation patterns are indeed reported to be subtype-specific [79] with a general increase in methylation of CpGs in luminal B tumors, which is not in keeping with the higher 2-HG levels reported in basal and HER2-enriched tumors in our case series. It was recently reported that DNA hypermethylation pattern across basal-like breast cancer does not correlate with tumor progression as it simply mirrors the repressed chromatin state of the tissue of origin. On the contrary, the hypermethylation pattern in the luminal subtype impacts on the gene expression pattern and possibly contributes to tumor progression and could therefore represent an actionable alteration [80].

### 4.3. Amino Acid Metabolism

These findings might have been indicative of altered glutaminolysis (the generation of *α*-KG from glutamine and GLU), which is a critical metabolic process for most tumors owing to aconitase mutation. However, whereas no significant differences across breast cancer molecular subtypes were reported for glutamine levels (*p* = 0.27, ANOVA), the GLU/glutamine ratio significantly varied across subtypes (*p* < 0.001, ANOVA) with the basal-like subtype mostly contributing to such differences (HER2-enriched versus basal, *p* = 0.01, luminal A versus basal *p* < 0.001, and luminal B versus basal *p* < 0.001, Tukey post hoc analysis).

Increased glutamine metabolism is another alternative source of energy for cancer cells, including breast cancer, and is thought to be a central metabolic pathway cooperating with glycolysis [81]. Metabolites derived from glutamine metabolism (NADH, glutathione, and ammonia) could be involved in the reduction-oxidation status in cancer cells and may lead to an increased tumor growth and drug resistance [82, 83]. *In vitro* studies have indeed shown that a high glutamine supply protected MCF7 cells from tamoxifen-induced apoptosis [82]. Immunohistochemical staining of breast cancer tissues indicates that HER2 positive and TNBC exhibit the more frequent expression of glutamine metabolism-related proteins than other subtypes [84].

In the literature, additional comparison studies on specific metabolic alterations in early breast cancer were done by comparing TNBC samples with triple-positive breast cancer (TPBC) samples. In a study using HR-MAS MRS, Cao et al. [85] show that TNBCs are characterized by lower glutamine levels and increased GLU levels compared to TPBC. The increased glutaminolysis metabolism in TNBC may represent a pathway worth to be targeted. Interestingly, the same study also reports increased glycine levels in HER2+ tumors, probably associated with their increased aggressiveness. Asiago et al. [86] observed that an elevated level of GLU was associated with disease outcome in breast cancer patients. The high GLU-to-glutamine ratio is found in breast cancer tissues as compared to normal tissues [87] and *in vitro* in highly invasive and drug-resistant breast cancer cells compared with noninvasive breast cancer cells [88].

The kynurenin pathway, linked to triptophane metabolism, has received increasing attention due to its connection with inflammation, immune system, and certain neurological conditions. Significantly different levels of kynurenin (*p* < 0.001, ANOVA) were detected across subtypes with an increase in basal-like (*p* < 0.001 basal-like versus luminal A, luminal B, and HER2-enriched, Tukey post hoc test) suggesting that activation of IDO, and consequently the development of inflammation and of immune tolerance, may represent a therapeutic target in such tumors (Figure 3(a)).

### 4.4. Lipid Metabolism

**Figure 4:**
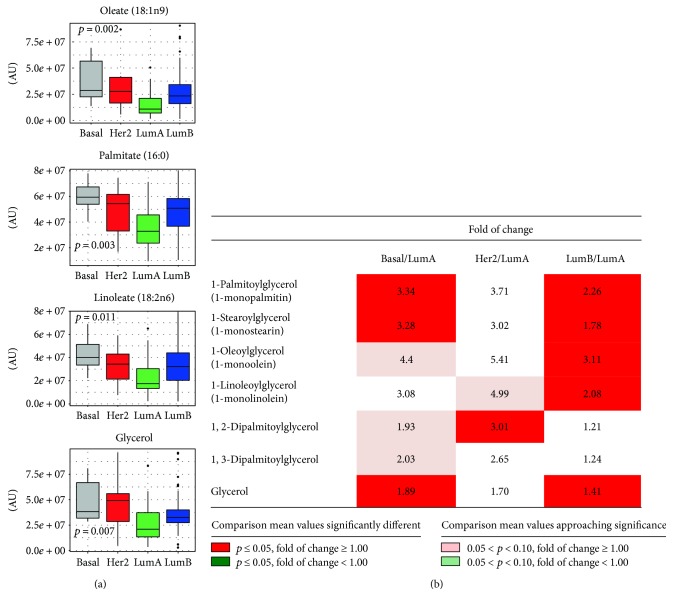
Diacylglycerol, monoacylglycerol, and fatty acid metabolism. (a) Box plots representing relative amounts of fatty acids and glycerol across molecular subtypes. *p* values refer to one-way ANOVA on raw data. (b) Relative differences in diacylglycerols and monoacylglycerols across molecular subtypes. Data for basal-like, HER2-enriched, and luminal B tumors, expressed as fold changes of raw data peak intensities with respect to the luminal A subtype, are reported for each metabolic intermediate. Statistical significance of differences between mean values for each reported comparison was tested by the Welch *t*-test. Color codes refer to the range of calculated *p* values and to the direction of the differences between means, as detailed in the figure inset. AU = arbitrary units.

Regarding lipid metabolism, we evaluated de novo biosynthesis, the intracellular accumulation, and catabolism. The level of numerous free fatty acids, including linoleate (*p* = 0.011, one-way ANOVA), palmitate (*p* = 0.003, one-way ANOVA), and oleate (*p* = 0.002, one-way ANOVA), was significantly different across all four subtypes (Figure 4). The accumulation of these lipids was also accompanied by an elevation in triacylglycerol catabolites such as glycerol (*p* = 0.01 one-way ANOVA) and monoacylglycerols (MAGs).

In particular, we found a significant accumulation of 1-MAGs in luminal B breast tissues as compared to other breast cancer subtypes. MAGs included both palmitic (C16) and stearic (C18) acyl chain with different degree of unsaturation.

The concentration of MAGs is regulated by the specific monoacylglycerol lipase (MAGL) and by glycerol-3-phosphate acyltransferase (GPAT) in de novo glycerolipid biosynthesis and diacylglycerol lipase (DAGL). Nomura et al. report an overexpression of MAGL expression in aggressive tumor cell lines and reveal that MAGL is part of a gene signature correlated with epithelial-mesenchymal transition and with stem-like properties of cancer cells [89, 90]. Expression of MAGL is often increased in cancer and promotes cancer pathogenesis, and the high level of 1-MAG observed in our luminal B tumors suggests a selective hydrolysis in the 2-position of the glycerol backbone with concomitant release of specific acyl chains (e.g., arachidonic acid), able to regulate a complex fatty acid network such as prostaglandine, lysophospholipid, and ether lipids, known to be involved in inflammation and tumor progression.

These findings suggest that in addition to de novo biosynthesis of lipids (potentially from citrate), higher fatty acid levels in the tissue may also be contributed by lipolysis. Fatty acids are a critical energy source that fuels oxidative metabolism and ATP generation. In addition to elevated free fatty acid levels, there was an accumulation of palmitoylcarnitine (*p* < 0.001, one-way ANOVA), stearoylcarnitine (C18) (*p* < 0.001, one-way ANOVA), and oleoylcarnitine (*p* = 0.001, one-way ANOVA) in luminal B, basal, and HER2-enriched breast cancer, suggesting the conjugation of long-chain fatty acids to carnitine for transport into the mitochondria and subsequent oxidation in such tumors.

**Figure 5:**
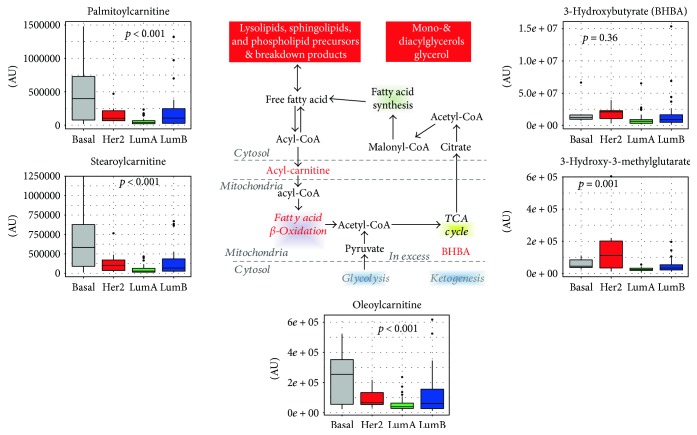
Fatty acid oxidation. Intermediates participating in fatty acid oxidation and their subcellular localization are reported. For metabolites indicated in red color, levels across breast cancer molecular subtypes are reported as box plots. *p* values refer to one-way ANOVA on raw data. TCA: tricarboxylic acid cycle; 3-HBA: 3-hydroxybutyric acid; AU = arbitrary units.

Whereas accumulation of fatty acids may suggest changes in synthesis or utilization as described above, a portion of fatty acids was potentially being oxidized as indicated by accumulation of the ketone body 3-hydroxybutyrate (3-HBA). 3-HBA is generated from excess acetyl-CoA often resulting from FAO, and it is an internal indicator of excessive lipid oxidation and of potential mitochondrial dysfunction. However, since levels of 3-HBA were comparable among subtypes, there was an indirect suggestion that FAO did not differ among breast cancer molecular subtypes (Figure 5). Together, these findings suggest that lipolysis and membrane biogenesis were enhanced in basal and HER2 breast cancers in agreement with previously published studies, thus demonstrating that overproduction of fatty acids facilitates tumor progression and cancer cell survival [91, 92]. The data also suggest that whereas luminal B tumors preferentially rely on fatty acids for energy, HER2-enriched and basal-like tumors show also alterations in glucose/glutamine metabolism. Since fatty acid synthesis and fatty acid metabolism, which have both been recognized as potential targets for cancer therapy in breast cancer [93], differ according to the molecular subtypes, appropriate treatment should be tailored accordingly [94].

Different breast cancer molecular subtypes seem to have different metabolic lipid signatures that are worth investigating before considering FAS as a strong therapeutic target. In such a context, our data could open a new treatment possibility for luminal B tumors.

### 4.5. Phospholipid Metabolism

**Figure 6:**
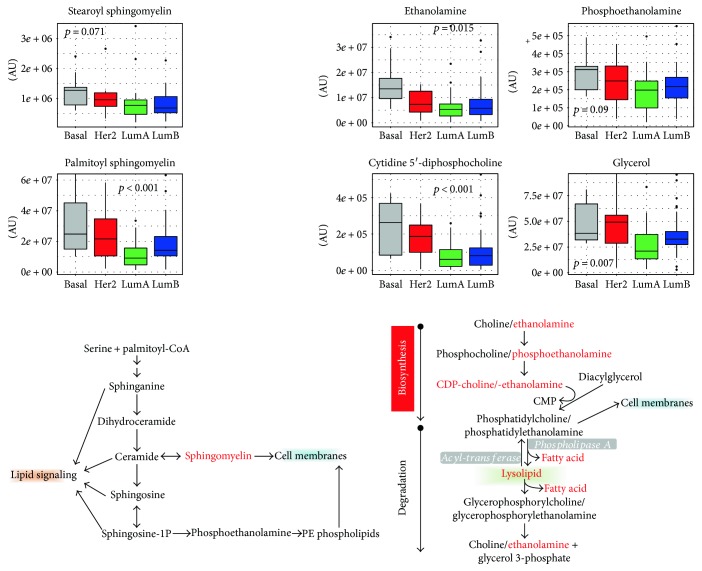
Phospholipid and sphingolipid metabolism. Intermediates participating in the phospholipid and sphingolipid metabolisms are schematically reported. For metabolites indicated in red-color fonts, levels across breast cancer molecular subtypes are reported as box plots. *p* values refer to one-way ANOVA on raw data. AU = arbitrary units.

Abundant fatty acid levels may suggest alterations in phospholipid metabolism. Regarding glycerol phospholipids, different levels of ethanolamine (*p* = 0.02, ANOVA), phosphoethanolamine (*p* = 0.09, ANOVA), cytidine-5′diphosphocholine (*p* < 0.001, ANOVA), DAG, and lysolipids suggest distinct membrane remodeling across subtypes. Similarly, different levels of sphingolipids such as palmitoyl sphingomyelin (*P* < 0.001, ANOVA) and to a lesser extent stearoyl sphingomyelin (*p* = 0.07, ANOVA), which were observed among subtypes (Figure 6), may also reflect a change in cellular membrane dynamics. These differences were greatest in basal-like and HER2-enriched tumors and potentially reflect a greater capacity of such tumors to grow.

Besides cellular membrane dynamics, phospholipid metabolism is also involved in intracellular signaling. There is a close network between oncogene-induced cell signaling through multiple postreceptor pathways and phospholipid metabolism. The phosphatidylinositol 4-phosphate 5-kinase I*γ* (PIPKI*γ*) is overexpressed in TNBC, and its loss has been shown to impair the PI3K/Akt activation in TNBC cells [95]. Furthermore, two major enzymes involved in the agonist-induced phosphatidylcholine cycle, that is, ChoK and PtdCho-specific phospholipase C (PLC), are overexpressed and differentially activated in various breast cancer subtypes, including TNBC, with implications on expression and oncogenic function of members of the EGF receptor family [96, 97]. This body of evidence suggests that enzymes involved in phospholipid biosynthesis and catabolism could act as key regulators of breast cancer progression.

### 4.6. Metabolomic Footprint in Breast Cancer

**Figure 7:**
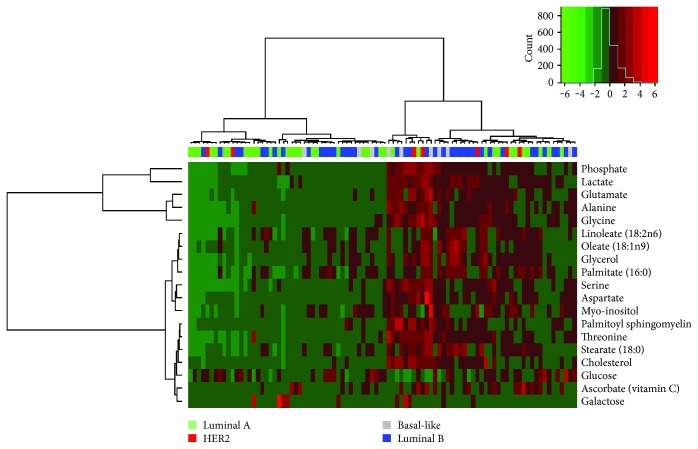
Metabolic clustering of breast cancer in relation to gene expression subtypes. Unsupervised metabolic cluster of the 19 metabolites with the highest interquartile range (above 95th percentile) for 92 primary breast tumors with Euclidian distance and Ward linkage. Each column represents a tumor, and each row represents a metabolite. The color legend refers to the molecular subtype as specified in the figure inset.

We finally explored whether the metabolite footprint of breast tumors is able to identify specific groups of tumors beyond classical molecular subtypes or within each specific molecular subtype. This was done by unsupervised clustering using the set of metabolites characterized by the highest variability across samples (evaluated in terms of interquartile range). Data are reported as a heat map in Figure 7.

Metabolite levels split the tumors into two groups characterized by high versus low levels of all of the specified metabolites, except for glucose whose levels where equally variable among the two clusters. Luminal B tumors were equally represented within the two clusters (45 versus 55%), whereas two-thirds of the luminal tumors fall into the low-metabolite level cluster in contrast to HER2-enriched and basal-like tumors which were mostly (69%) represented in the high-metabolite level cluster.

## 5. Treatment Implications and Future Research Strategies

Deregulated metabolic pathways in cancer such as glycolysis, the Krebs cycle, mitochondrial respiration, glutaminolysis, and FAO are possible drug target candidates.

Many studies, including research programs supported by pharmaceutical companies, have focused on the development of inhibitors targeting glycolytic pathways.

Main drugs targeting metabolism used in clinical and preclinical studies are reported and discussed in Table 2.

Based on the metabolic differences that we have reported among molecular subtypes, targeting glycolysis might represent a possible approach for HER2-enriched and basal-like tumors where this pathway is activated compared to luminal tumors. In such a context, the first strategy which can be adopted consists in the employment of glucose analogs such as 2-deoxyglucose, which enter the cell via glucose transporters and are phosphorylated by hexokinase, which cannot be further metabolized. Consequently, 2-deoxyglucose-6-phosphate is accumulated triggering inhibition of glycolytic enzymes and of glucose catabolism. Although many studies have reported the efficacy of this compound, either alone or in combination with other anticancer drugs, or with local treatments (surgery or radiations), both the preclinical and the clinical studies have pointed out high toxicity as a strong limit of such an approach [98].

Other strategies, explored at the preclinical level, were instead directly targeting specific isoforms of the glycolytic pathway, increasing the drug specificity towards cancer cells and limiting toxicity to normal cells. In fact, targeting the activity of 6-phosphofructo-1-kinase (PFK-1), the rate-limiting step of glycolysis by 3PO/PFK158 inhibitors of 6-phosphofructo-2-kinase/fructose-2,6-biphosphatase 3 (PFKFB3), directly affects the entire glycolytic pathway in different preclinical models [99].

At the end of glycolysis, the generation of lactate from pyruvate catalyzed by LDH replenishes NAD^+^ necessary for enhanced glycolytic flux in the tumor and is responsible for extracellular acidification, leading to activation of metalloproteinases. The overexpression of a specific LDH isoform, the LDH-A, whose expression is regulated at transcriptional level by MYC and HIF-a, has been observed in several tumor types [100, 101]. Indeed, silencing of LDH-A in tumor cells by siRNA reduced tumor growth *in vitro* and *in vivo* suggesting that LDH-A may represent an effective antitumor therapy target [102]. Consistently, the pharmacological inhibition of LDH-A by the natural phenol gossypol was approved in clinical studies [103].

Lactate is extruded into extracellular medium by a family of monocarboxylate transporters (e.g., MCT4) and can be imported by the isoform MCT1 for being used in the TCA cycle by neighboring cells (whose oxidative metabolism is predominant as compared to glycolytic metabolic cells). Different studies suggest that MCT1 may be an effective target for therapeutic intervention of the glycolytic tumor, because by blocking lactic acid import into aerobic cells, such cells take up glucose leaving the anaerobic cells to die due to glucose deprivation [104].

Many preclinical studies have shown that enzymes involved in the TCA cycle and mitochondrial respiration could represent targets for the development of novel anticancer drugs [105]. The GLU metabolism is modified in breast cancer with a general increase in GLU and 2-HG, and levels of these metabolites are even further increased in steroid hormone receptor-negative compared to hormone receptor-positive tumors. GLS, the enzyme catalyzing the conversion of glutamine to GLU, which can enter the mitochondrion and the TCA cycle, represents a possible drug target since glutaminase inhibitors are available and are tested in clinical trials [106]. Further, the GLU to glutamine ratio may represent a valuable biomarker for selecting patients for therapeutic inhibition of GLS [87].

Other therapeutic opportunities for selected breast tumors, as for example luminal B, which rely preferentially on fatty acid metabolism for energy production, are in the lipid metabolism. Indeed, Hilvo et al. [107] have shown that lipid metabolism is an attractive target for antitumor drugs, as it not only differs between normal and tumor tissue but also varies among tumor subtypes and is correlated with aggressiveness. Tumors are characterized by increased levels of palmitate-containing PtdCho, and other products of de novo fatty acid synthesis with respect to normal tissue and activation of lipid metabolism represent a well-known feature of malignant transformation (which is known by the name of lipogenic phenotype [108]). Higher product levels of deriving from de novo fatty acid synthesis were reported for ER− than in ER+ tumors and for grade 3 tumors.

Lipogenesis which is fueled by pyruvate and glutamine represents the anabolic program of the tumor cell promoted by the Warburg effect, and as such many enzymes of the lipogenic cascade have been the object of attempts to develop specific inhibitors with potential anticancer activity. However, as recently reviewed by Kinlaw et al. [93], breast tumors not only synthesize fatty acids but also present an increased uptake of fatty acids supported by the expression of *LPL* and *CD36*, which suggests that uptake could also be targeted by specific inhibitors.

Only little attention has been devoted to developing inhibitors against FAO. The agent etoximir inhibitor of CPT1, responsible for mitochondrial import of fatty acids mediated by the carnitine shuttle, decreases intracellular ATP levels as well as cell viability in glioblastoma [109] and affects tumor growth in preclinical models [110].

New antitumoral approaches are based on the blockade of immune-inhibitory pathways such as the inhibition of the IDO pathway. The rate-limiting enzyme IDO catalyzes the first reaction in the tryptophan degradation and plays an important role in cancer progression as well as in cancer initiation. It has been indeed described to support inflammation in the tumor microenvironment and has an immunomodulatory role as it is involved in the suppression of T and of NK cells and in generation and activation of Tregs and myeloid-derived suppressor cells [111, 112]. IDO activation is stimulated by inflammatory cytokines, such as IFN-*γ* and TNF-*α*. Consequently, IDO represents an interesting therapeutic target for many tumors including breast cancer and a phase II clinical trial (NCT01792050) in HER2-negative metastatic breast cancer patients in combining the IDO inhibitor Indoximod (NLG2101) with docetaxel which is currently ongoing.

Insight, such as that reported here, on the correlation between subtypes and metabolites of these crucial pathways is therefore particularly important. Despite there is a lot of evidence at the preclinical level of a partial antitumor activity of IDO inhibitors, there might be a need to apply combined strategies against multiple immune-inhibitory mechanisms present in the tumor microenvironment concurrently such as PD-1/programmed cell death ligand-1 (PD-L1) or cytotoxic T-lymphocyte-associated protein 4 (CTLA4) signaling, for obtaining an optimal therapeutic effect [113].

The relevance of targeting metabolic pathways is also supported by data linking metabolic profiles to clinical outcome, with lower concentrations of glycine in patients with good prognosis compared to those with bad prognosis [114]. In locally advanced breast cancer treated with doxorubicin, a decrease in glycerophosphocholine predicted long-term survival and response to treatment [115]. Higher levels of glycine and lactate were found to be associated to lower survival rates in ER+ patients [116], whereas opposite trends were reported by Cao et al. [117] in the context of neoadjuvant treatment. Consistently, depletion of amino acids activates mTOR signaling and reduces protein translation with subsequent proliferative arrest in cancer cells [118].

Finally, different inhibitors were developed in experimental models against several enzymes in lipid biochemistry including FASN, ACCs, ChoK, and cholesterol pathways [119].

The importance of targeting metabolism is further supported by well-known associations between metabolic diseases (obesity, hyperglycaemia, hyperlipidaemia, and insulin resistance) and the increased risk of developing various types of cancer or poor prognosis in affected cancer patients. In fact, some metabolic drugs such as metformin (a biguanide that is generally used for the treatment of type 2 diabetes) and statins (inhibitors of cholesterol synthesis) are known to reduce cancer-related morbidity and mortality [120–123].

This scenario suggests that these drugs act in the complex network signaling among metabolic pathways that include several biochemical mechanisms at the receptor level (e.g., insulin-like growth factor 1 (IGF1)), metabolic checkpoint (5′ AMP-dependent protein kinase (AMPK) activation), and anabolic/catabolic metabolism, able to modulate cancer proliferation and tumor-promoting inflammatory pathways.

The metabolic reprogramming of malignant cells offers therefore a large number of potential drug targets, but only by careful dissection of metabolic processes, and by identifying links between putative metabolic targets and specific tumor subtypes, we will be able to translate this opportunity into the development of novel anticancer agents to treat breast cancer.

## Figures and Tables

**Table 1:** Number of biochemicals with statistically significantly (*p* < 0.05^∗^) different levels at pair-wise comparison between molecular subtypes.

	Basal	HER2-enriched	Luminal A
HER2-enriched	15	—	—
Luminal A	110	11	—
Luminal B	34	16	85

^∗^Welch's two-sample *t*-test.

**Table 2:** Potential therapeutic drugs targeting metabolic enzymes of cancer.

Target	Drug	Study phase
Glucose transporters	Phloretin	Preclinical
	2-Deoxyglucose	Phase 1

Hexokinase	2-Deoxyglucose	Phase 1
	Lonidamine	Clinical trial
	3-Bromopyruvate	Preclinical

Fructose-2,6-bisphosphatase isozyme 3 (PFKFB3)	3-(3-Pyridinyl)-1-(4-pyridinyl)-2-propen-1-one (3PO)	Preclinical

Pyruvate kinase M2 (PKM2)	TLN-232/CAP-23	Phase 2

Pyruvate dehydrogenase kinase 1 (PDK1)	Dichloroacetic acid (DCA)	Phase 1

Monocarboxylate transporter-1 (MCT1)	AZD3965	Phase 1/2

Mitochondrial complex 1	Metformin	Clinical trial
	Phenformin	

Carnitine palmitoyltransferase-1 (CPT-1)	Etomoxir	Tested in *clinical trials*; retired owing to hepatotoxicity

Choline kinase	CK37	Preclinical
	TCD-717	Phase 1

HMG-CoA reductase (HMGCR)	Statins	Nononcologic clinical trial

Asparagine	L-asparginase	Phase 2

Arginine	Arginine deaminase	Phase 2

Nicotinamide phosphoribosyl transferase	FK866/APO866	Phase 2

Isocitrate dehydrogenase (IDH)	AGI-5198	Preclinical
	AGI-6780	

Indoleamine-2,3-dioxygenase (IDO)	INCB 024360	Phase 1/2
	Indoximod (NLG2101)	

## Abbreviations

2-HG: 2-Hydroxyglutarate
3HBA: 3-Hydroxybutyrate
ACC: Acetyl-CoA carboxylase
a-KG: *α*-Ketoglutarate
AMPK: 5′ AMP-dependent protein kinase
ARG: Arginase
ChoK: Choline kinase
CPT1: Carnitine O-palmitoyltransferase 1
CTLA4: Cytotoxic T-lymphocyte-associated protein 4
DAG: Diacylglycerol
DAGL: Diacylglycerol lipase
F1,6BP: Fructose-1,6-bisphosphate
FAO: Fatty acid oxidation
FASN: Fatty acid synthase
^18^F FDG: Flurodeoxyglucose F 18
FH: Fumarate hydratase
GC: Gas chromatography
GC-TOF-MS: Gas chromatography time-of-flight mass spectrometry
GLS1: Glutaminase
GLU: Glutamate
GLUT-1: Glucose transporter 1
GPAT: Glycerol-3-phosphate acyltransferase
GPI: Glycosylphosphatidylinositol
HR-MAS: High-resolution magic angle spinning
IDH: Isocitrate dehydrogenase
IDO: Indoleamine-2,3-dioxygenase
IGF1: Insulin-like growth factor 1
iNOS: Nitric oxide synthase
LC: Liquid chromatography
LDH: Lactate dehydrogenase
LDHA: Lactate dehydrogenase-A
MAG: Monoacylglycerol
MAGL: Monoacylglycerol lipase
MCD: Malonyl-CoA decarboxylase
MS: Mass spectrometry
NMR: Nuclear magnetic resonance
PC-PLC: Phosphatidylcholine-specific phospholipase C
PD-L1: Programmed cell death ligand-1
PFK-1: 6-Phosphofructo-1-kinase
PFKFB3: 6-Phosphofructo-2-kinase/fructose-2,6-biphosphatase 3
PGC-1*α*: Peroxisome proliferator-activated receptor gamma coactivator 1-alpha
PHD: Prolyl hydroxylase
PIPKI*γ*: Phosphatidylinositol 4-phosphate 5-kinase I*γ*
PLA2: Phospholipase A2
PLC: Phospholipase C
PLD: Phospholipases D
PPAR: Peroxisome proliferator-activated receptors
PPP: Pentose phosphate pathways
PtdCho: Phosphatidylcholine
Ptdlns: Phosphatidylinositol
SCD: Stearoyl-CoA desaturase
SDH: Succinate dehydrogenase
SREBF1: Sterol regulatory element-binding transcription factor 1
TCA: Tricarboxylic acid
TDO: Tryptophan-2,3-dioxygenase
TNBC: Triple-negative breast cancer
TPBC: Triple-positive breast cancer
TPH-1: Tryptophan hydroxylase-1.

## Supplementary Materials

Supplementary Materials Raw data on metabolites.
